# The Locus Coeruleus in Chronic Pain

**DOI:** 10.3390/ijms25168636

**Published:** 2024-08-08

**Authors:** Jorge Castejón España, Anusha Yasoda-Mohan, Sven Vanneste

**Affiliations:** 1 Lab for Clinical and Integrative Neuroscience, Trinity College Institute for Neuroscience, School of Psychology, Trinity College Dublin, D02 PN40 Dublin, Ireland; castejnj@tcd.ie (J.C.E.); anusha.mohan@tcd.ie (A.Y.-M.); 2 Compass Physio, A83 YW96 Enfield, Ireland; 3 Global Brain Health Institute, Trinity College Dublin, D02 PN40 Dublin, Ireland; 4 Brain Research Centre for Advanced, International, Innovative and Interdisciplinary Neuromodulation, 9000 Ghent, Belgium

**Keywords:** chronic pain, locus coeruleus, comorbidities, pain pathways, pain inhibition

## Abstract

Pain perception is the consequence of a complex interplay between activation and inhibition. Noradrenergic pain modulation inhibits nociceptive transmission and pain perception. The main source of norepinephrine (NE) in the central nervous system is the Locus Coeruleus (LC), a small but complex cluster of cells in the pons. The aim of this study is to review the literature on the LC-NE inhibitory system, its influence on chronic pain pathways and its frequent comorbidities. The literature research showed that pain perception is the consequence of nociceptive and environmental processing and is modulated by the LC-NE system. If perpetuated in time, nociceptive inputs can generate neuroplastic changes in the central nervous system that reduce the inhibitory effects of the LC-NE complex and facilitate the development of chronic pain and frequent comorbidities, such as anxiety, depression or sleeping disturbances. The exact mechanisms involved in the LC functional shift remain unknown, but there is some evidence that they occur through plastic changes in the medial and lateral pathways and their brain projections. Additionally, there are other influencing factors, like developmental issues, neuroinflammatory glial changes, NE receptor affinity and changes in LC neuronal firing rates.

## 1. Introduction

Pain is defined by The International Association for the Study of Pain (IASP) as “an unpleasant sensory and emotional experience associated with or resembling that associated with actual or potential tissue damage” [1]. When acute pain (the “unpleasant sensory and emotional experience”) is perpetuated in time, under specific contextual circumstances (emotional distress, genetic predisposition, childhood emotional trauma, etc.), it can evolve into a complex multifactorial syndrome named chronic pain [2]. The IASP defines it as any pain that persists or recurs for more than 3 months and subdivides it into primary and secondary, with six different subcategories for the latter (neuropathic, cancer, visceral, etc.) [2,3]. It has a prevalence of approximately 20% around the world, and its frequent clinical presentation has both mental (anxiety, depression and other cognitive impairments) and medical comorbidities (diabetes type II, hypertension, fatigue, etc.) that account for a substantial financial burden for health systems across the globe [4,5,6,7].

Pain is a survival strategy that provides animals with a signal of internal or external threats. It has an evolutionary advantage as, for example, individuals suffering from congenital analgesia rarely survive their third decade of life [8]. Interestingly, the same nociceptive input could be perceived as painful depending on the environment, the temporal summation of its nociceptive signal or the individual’s emotional state. This has been observed for centuries by physicians in war zones and was precisely depicted by an anesthesiologist in World War II [9]. Later research confirmed these clinical observations and concluded that pain perception is not correlated to the damage inflicted on the body [10].

Physiologically, the perception of a painful stimulus relies on four hierarchical mechanisms: transduction, transmission, modulation and perception [11]. Using them as a reference, research has proposed terms such as peripheral sensitization (transduction and first-order sensory neuron alterations) and central sensitization (transmission and modulation of the signal at the central nervous system), attempting to explain the mechanisms that could sustain chronic pain beyond the natural history of a given injury [12]. However, these ideas have recently been challenged as the unique drivers of chronic pain perpetuation [13].

More recent theories indicate that chronic pain might emerge from a central imbalance between activation (nociceptive transmission) and inhibition (modulation via norepinephrine (NE), serotonin (SE) or dopamine), which have also been associated with the three well-established functional neural networks (default mode, executive and salience networks) [14,15,16].

In the central nervous system, NE is produced by seven clusters of cells located in the brainstem [17,18]. Of these seven groups of cells, the A6 or Locus Coeruleus (LC) is the sole source of NE for the cortex and has accumulated most of the research in the last four decades [19]. To date, the LC has been associated with pain inhibition and other cognitive functions, such as attention, memory, arousal and sleep [20,21,22]. Furthermore, there is sufficient evidence to suggest that the LC might also play an important role in chronic pain establishment and perpetuation, through its interaction with the ascending nociceptive pathways and the descending inhibitory pathways [23,24,25,26].

Therefore, this narrative review [27] aims to integrate the current state of the art of A6 or LC within the latest neuroscientific chronic pain models, focusing on its influence on a variety of chronic pain clinical presentations and comorbidities. First, the nociceptive pathways will be presented, followed by an exploration of the LC and its interaction with these pathways. Then, the intricate connections between the LC and different comorbidities associated with pain, such as anxiety, depression and sleep disorders, will be delved into. Finally, the role of the LC in the process of pain chronification and its clinical implications will be examined.

## 2. Nociceptive Pathways

The basic ascending neuroanatomical pathways implicated in the conduction of nociceptive signals and their context are well established. Among these tracts, the two major pathways are the direct lateral spinothalamic tract, which projects to the ventral posterolateral nucleus of the thalamus and carries the duration, intensity and location of the pain, and the indirect medial spino-reticular-thalamic tract projects to the medial thalamus and carries the unpleasantness of the stimulus. The former carries superficial precise pain signals, whereas the latter contains visceral unspecific pain signals. Complementarily, other tracts also play a role in the contextualization of a noxious stimulus (i.e., spino-tectal or spino-reticular tracts). Several books have described these tracts in detail [28,29,30,31].

**Figure 1:**
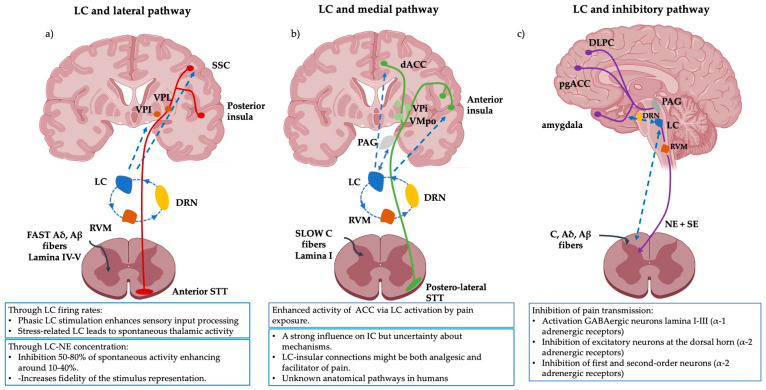
Schematic representation of the pain pathways and the known modulatory effects of the LC on them. (**a**) The lateral pathway is influenced by the LC via the SSC and the thalamus. The LC directly modulates the intensity, localization and type of pain perception. (**b**) The medial pathway processing with its insular and ACC projections is enhanced via the LC but the anatomical pathways and the neurophysiological mechanisms implicated in humans remain unknown. (**c**) The inhibitory pathway is broadly influenced by LC activity and vice versa. The inhibitory signal is strongly modulated by LC activity and its direct projections to the amygdala. The amygdala, in conjunction with the pgACC and the DLPF modulates descending LC-NE inhibitory pathways. Several investigations suggest that CP might be explained as a lack of inhibition or an imbalance between inhibition output and ascending inputs. Abbreviations: LC: Locus Coeruleus, VPL: ventroposterior lateral nucleus, VPI: ventroposterior inferior nucleus, VMPo: ventromedial posterior pars oralis, SSC: somatosensory cortex, dACC: dorsal- anterior cingulate cortex, PAG: periaqueductal grey, RVM: rostral ventral medulla, DLPC: dorsolateral prefrontal cortex, pgACC: pregenual anterior cingulate cortex, DRN: dorsal raphe nucleus, SE: serotonin. This figure was created using PowerPoint (version 16.87) and BioRender 2024.

In brief, when a mechanical stimulus activates the free nerve endings of the primary afferent neurons, it is transduced from mechanical to electrochemical and transmitted from the periphery to the central nervous system through Aδ, Aβ (myelinated) and C fibres (non-myelinated). After synapsing with the secondary afferent neurons at the dorsal horn, the signal continues upstream mainly through the spinothalamic tracts (medial and lateral) and reaches different regions of the thalamus. Once in the thalamus, the original mechanical stimulus now has different subcomponents that contain features of the painful signal, such as context, location and intensity. From the thalamus, signals project through different pathways to the prefrontal cortex (PFC; Brodmann area (BA) 46, 9, 10), the somatosensory cortex (SSC; BA 1–3, 5, 7), the insula and the anterior cingulate cortex (ACC; BA 32), where the subjective perception of the nociceptive input is generated [32,33] (Figure 1).

The modulation of all ascending noxious sensory signals relies on three different phenomena that occur at various levels of the central nervous system: the segmental modulation that is based on fibre impulse conduction and spinal inhibitory neurons [34,35,36,37]; the endogenous opioid system that modulates the nociceptive signal through opioid (endorphins, enkephalins and dynorphins) activation of specific opium derivative receptors [38]; and the descending inhibitory pathway that releases SE and NE at the dorsal horn of the spinal cord.

This latter inhibitory pathway originates at higher hierarchical levels of the nervous system and descends towards the dorsal horn of the spinal cord, relying on the following neuroanatomical hubs in a rostral to caudal order: the dorsolateral PFC (BA 9), the pregenual ACC (BA 33), the reticular nucleus of the thalamus, the amygdala, the periaqueductal gray (PAG), the rostroventral part of the medulla oblongata, the noradrenergic groups in the brainstem (A1–A7) and the posterior and anterolateral medulla. Here, inhibitory interneurons release opioids, such as beta-endorphins, leu-enkephalins and dynorphins [17,28,31,38]. This inhibitory system is mainly regulated by NE, and it is usually named descending noradrenergic pain modulation [17].

In short, pain perception is mainly based on three independent but interconnected anatomical pathways: lateral or direct, medial or indirect, and descending or inhibitory (NE-SE mediated). Neither of them accounts for the full perception of pain, but a dysfunction in any of them will modify the perception of a given nociceptive signal. Interestingly, diverse chronic pain conditions have recently been explained as an imbalance between pain input and suppression [14,39,40]. Based on these well-established anatomical principles, the following sections will depict the potential modulatory role of the LC in each of the three pain pathways and their relevant clinical implications.

### Practical Example: From Caesarean Section to Emotion

Based on these neuroanatomical pathways, when the signal of a multi-layered wound such as a Caesarean section reaches the thalamus, it comes decomposed in two different nociceptive signals. Before their arrival into the thalamus, they carry raw nociceptive information that could be characterized as painful at higher hierarchal levels based on the described anatomical pathways:

- The lateral pathway (painfulness) through its Aδ, Aβ myelinated fibres will provide features of pain perception such as location or intensity thanks to the representation of the lower abdominal area in the SSC [30,31,32].
- The medial pathway (suffering) via its unmyelinated C fibres will carry the context of the pain as unconscious, interoceptive, unspecific visceral pain on the visceral insular cortex (BA 13, 16). The visceral afferents project to areas associated with proprioception and emotional processing, such as the insula, thalamus, ACC or PFC [41]. It could also escalate into more complex perceptions such as memories or emotions based on the context [29,32,33,42].

Finally, both nociceptive signals will be integrated with the internal or external contexts and previous experiences in associative areas such as the anterior insular, parietal or frontal cortices [33,43,44]. That combination of perception, context and memory represents the full experience of pain from a single injury [14,45].

**Figure 2:**
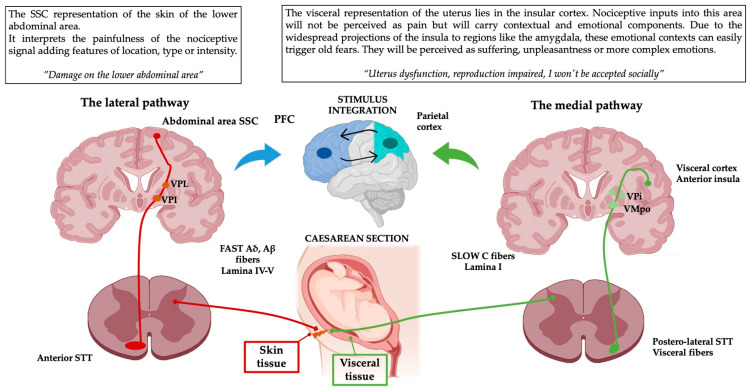
Schematic representation of the pain pathways on a practical example. This simplification of the complexity of pain perception clearly illustrates how a simple multi-layered injury can have different subcomponents that contribute to the total pain experience emerging from it. The widespread projections of medial and lateral pathways will end up integrating the processed nociceptive signals with previous memories and existing environmental or internal contexts. These contexts are perceived through other emotional triggering domains such as vision, audition, olfaction or interoception. PFC: prefrontal cortex, VPI: ventroposterior inferior nucleus, VMPo: ventromedial posterior pars oralis, PFC: prefrontal cortex, VPL: ventroposterior lateral nucleus, VPI: ventroposterior inferior nucleus, STT: Spinothalamic tract. This figure was created using PowerPoint (version 16.87) and BioRender 2024.

Given the high prevalence of physical and psychological Caesarean section associated disorders [46,47], it is important to approach these multi-layered scars using their complex neuroanatomical representation as a reference and not just as an aesthetic issue. This example aims to illustrate the importance of clinicians understanding the ascending pain pathways when prescribing treatments to patients with chronic pain (Figure 2).

## 3. Locus Coeruleus

The LC, also called the blue spot (due to the bluish colour of its neuromelanin), is a bilateral nucleus of approximately 20,000 neurons located adjacent to the fourth ventricle near the ponto-mesencephalic junction. It contains a relatively small amount of big neurons that are structured in a “core” where most cell bodies are located and a prominent “crust” named peri coeruleus where glial cells and GABAergic neurons intersperse with LC dendrites [22,48,49,50]. This nucleus has a unique gene expression profile with at least 3000 genes, of which 100 have significant gender-related differences in mammals [51]. For further information about gender-related differences in rodents, the reader is referred to the following references [52,53,54].

LC neurons’ firing rates oscillate from null activity in REM sleep to 0.5–5 Hz in different states of arousal and attention. From an electrophysiology perspective, the LC fires at an oscillatory rate of phasic (arousal, attentive state) and tonic activity (shifting attentional state) that resembles the Yerkes-Dodson arousal curve [55,56]. These fluctuations in activity could be spontaneous but are usually influenced by widespread connections with other regions of the central nervous system [22,55].

Histologically, the LC has traditionally been divided into four quadrants (i.e., rostral, caudal, ventral and dorsal) [22,48,57]. Different studies have proposed different functions for each subpopulation of neurons. In brief, the caudal LC has been associated with spinal cord projections, whereas the rostral LC is believed to project to and receive inputs from the telencephalon, diencephalon and mesencephalon. The ventral LC is proposed to take a more prominent role in antinociception or pain inhibition, whereas the dorsal LC would facilitate pain perception at the SSC [25,58]. Nevertheless, further research is needed to confirm or deny this functional subdivision and its potential clinical implications.

The LC is the sole source of NE for the cortex, and it is also released down the spinal cord to participate in different modulatory roles. NE is both a hormone and a neurotransmitter, whose main role is to alert and prepare the body to act by activating complex neurophysiological pathways [59,60]. The transport of NE relies on the concentration of a specific norepinephrine transporter encoded by the gene SLC6A2 [18,61]. The release of NE by the LC axons follows two different mechanisms: the classic specific “wiring transmission” where NE is released from vesicles at the axon terminal and the unspecific “volume transmission” that releases NE in a paracrine or local hormonal effect style [62]. The mere presence of NE does not explain the function of a given network, because that depends almost exclusively on the location and distribution of adrenergic receptors. These receptors could be classified into α1 (A, B, D), α2 (A, B, C, D), β1, β2 and β3 (see Table 1). Depending on their concentration, distribution and temporospatial summation in each context, NE could exert opposite effects in different situations. Given the importance of adrenergic receptors, the reader is encouraged to delve into further manuscripts to fully comprehend the complexity of their role [63,64,65,66].

It is important to note that most of the projections and cognitive functions associated with the LC-NE system are also targets of the neuromodulation exerted by other neuronal clusters. They include the ventral tegmental area, which is the main concentration of dopaminergic neurons in the human brain; the prepositus hypoglosi that inhibits LC activity through the release of GABA, the rostro ventral lateral medulla (RVM), which acts as an intermediate synapse centre for most of the painful stimuli that ascend through the spine; or the dorsal raphe nucleus, which contains 165,000 serotonergic neurons [49,61,67,68,69,70]. Some examples of these interactions include how the LC-derived excitatory synaptic activity inhibits SE release at the dorsal raphe nucleus through pre-synaptic α2 adrenergic receptors or the RVM excites the LC by releasing glutamate as a result of its ON and OFF neural circuits [71,72]. The reader can find an elegant atlas of the structural connectivity of these clusters in a research article by Levinson et al. (2023) [73].

Our current understanding of the projections of the LC relies largely upon animal models. Research on the LC and its projections in humans has encountered numerous limitations that need to be addressed for further advances. Some of the limitations of studying the LC come from the following: the small and heterogenous number of neurons existing on the nucleus together with a wide varicose-type ramification and widely spread branching throughout the central nervous system [22]; the lack of proper tools to directly measure the activity of the LC (3–4 mm wide and 1.5 cm long); the heterogeneity of neurons and glia present within the LC complex; or the complex and plastic interconnections with other nuclei of the brain stem. Optogenetics, advanced fMRI analysis or relatively new tools such as pupillometry analysis might help future researchers in the area [55,63,74,75].

## 4. LC and Its Connections with Pain Pathways

Studies have shown that chronic pain conditions are associated with alterations in LC neuron activity and NE release. These changes may contribute to the development and maintenance of chronic pain by altering the balance between pain inhibition and facilitation within the central nervous system.

The LC is also involved in modulating the affective and emotional aspects of pain. Dysregulation of the LC-NE system can influence emotional responses to pain, such as anxiety and depression, which are commonly comorbid with chronic pain conditions. This emotional component of pain can further exacerbate the overall pain experience and contribute to the chronicity of pain symptoms.

As depicted above, most of the current information about the LC in human beings is based on post-mortem anatomical and mammalian studies. Even though it is widely accepted that these models represent the LC in humans, statements about LC stem from animal model studies, and their clinical implications should be taken carefully.

What has been shown consistently is that the LC contributes to a variety of functions (attention, arousal, memory consolidation, etc.) that are possible due to its selective efferent connections, its widespread inputs and its internal functional organization [50,75,76]. The LC functional organization relies on anatomy, physiology, glial activity and spatiotemporal summation [22,48,57]. It might explain how a single source of inputs can target a cascade of network activation or how a temporal summation of independent stimuli might be sufficient to activate other neural circuitry [63,77,78].

Due to its location, widespread connections and neurophysiological properties, this small but complex nucleus might play an essential role in the regulation of the three neuroanatomical pathways described before. Next, this review integrates our current understanding of the LC projections with the widely accepted pain nociceptive pathways. Due to limitations in the literature mentioned above, only the known projections and their potential clinical implications will be focused on. It is also important to note that most of the LC connections are likely to be bidirectional, with feedback inhibitory and excitatory loops interacting in an intricate continuum. For further reading on this topic, the reader is referred to these articles [22,58,59,60]. Figure 1 aims to support the comprehension of this section.

### 4.1. Lateral Nociceptive Pathway and LC

The direct or lateral pain pathway processes the nociceptive input from Aβ, Aδ fibres that ascend from the laminas IV-V of the dorsal horn, through the contralateral anterior spinothalamic tract, towards the SSC. It encodes painfulness and different components of pain such as intensity, type (burning, aching, soreness, etc.) and localization, thanks to the involvement of the somatosensory and the associative parietal cortices [58,79,80].

The LC exerts a global influence on the final painfulness perception by inhibiting spontaneous activity on the targeted SSC areas (50–80% across the whole cortex) and enhancing the firing rate of others (10–40% on deeper layers of the cortex) and the overall fidelity of the stimulus representation [20,81]. This effect is mediated by NE concentration, and it is believed to be strongly influenced by phasic-tonic temporal profiles [20,26,82,83].

The exposure to persistent pain generates neuroplastic changes in sensory neurons of the SSC [84,85]. Due to differing temporal dynamics of neuroplasticity across various animal models and strain-specific variances in noradrenergic pain pathways, it remains difficult to generate direct evidence of the role of LC in these neuroplastic changes. However, the role of the LC in altering SSC functioning is currently being explored from different perspectives such as predictive coding [86,87] or a novel multidomain pathway model regarding sleep-related disturbances [88,89]. 

Studies involving the selective destruction of noradrenergic neurons have shown a reduction in neuropathic pain, and lidocaine administration to the LC contralateral to the lesion has mitigated evoked pain responses in specific rat strains [90]. However, similar lidocaine administration to the ipsilateral LC did not alter sensory hypersensitivity. Conversely, experiments utilising DREADDs (Designer Receptors Exclusively Activated by Designer Drugs) or lidocaine blockade of the LC, whether ipsilateral or contralateral, modulated neuropathic pain [91].

The direct effects of the LC on other hubs of the lateral pathway, such as the somatosensory thalamus (ventral posterolateral and ventral posteroinferior nucleus), have been less studied. However, LC firing rates might exert diverse modulatory effects on the thalamus both at the single-cell and global levels. In that way, under normal circumstances, phasic LC stimulation seems to enhance stronger sensory inputs, while stress-related LC activation leads to reduced evoked responses and more spontaneous activity [20,92,93]. This seems to be applicable for both the lateral and the medial pathways and has an important clinical relevance associated with frequent chronic pain comorbidities such as anxiety and depression.

### 4.2. Medial Nociceptive Pathway and LC

The indirect or medial pain pathway processes nociceptive signals from the non-myelinated C fibres that ascend from the lamina I through the lateral spinothalamic tract. It encodes suffering or unpleasantness due to its main cortical hubs: the rostral to the dorsal ACC (BA 24, 32) and the anterior and posterior insular cortices [14,94]. Interestingly, cumulative evidence suggests that emotional and nociceptive inputs are integrated through anterior and posterior insular mutual connections and their ACC projections [95,96,97].

Aside from the LC-NE cortical innervation of these regions, rodent models have shown that ascending LC projections to the ACC increase glutamatergic transmission, facilitating contextual, sensory and nociceptive processing [23,98,99]. In addition, the anterior insula’s role in emotional processing might also be influenced by the LC, but there is more uncertainty about how it might exert its influence [33,100]. What has been proven is that inhibitory projections from the insula into the ACC modulate the affective but not the sensory components of neuropathic pain perception [101].

Studies on animal models indicate that excessive activation of the insula contributes to hyperalgesia and pain perception through a descending facilitatory pathway that has not yet been described in humans [33,97]. These ideas position the anterior insula as a potential chronic pain driver under pathological circumstances that might be facilitated by LC-NE release. It is also important to note that the insula is an essential component of the salience network, which is very dependent on the context and has been frequently linked with suffering [100,102]. The LC might enhance or mitigate pain-related suffering by its influence on contextual evaluation areas through its connections to the salience network and other sensory and nociceptive processing areas. Further research is needed to elucidate the exact neuroanatomical pathways implicated in the LC regulation of suffering in humans.

### 4.3. Descending Inhibitory Pathway, Central Autonomic Network and LC

The inhibitory pathway balances the medial and lateral input signals and is the main player responsible for pain reduction effects such as placebo or analgesia [103,104].

The origin of the inhibitory signal is still under debate, but it can be influenced by areas that govern cognitive, emotional and motivational processes, such as the dorsolateral PFC, orbitofrontal cortex (BA 11, 12), the pregenual ACC, the anterior insula or the amygdala [82,105,106,107]. These areas can exert a modulatory role on the descending pain pathway but are not the source of the neurotransmitters that will modulate the nociceptive input. Instead, they act as orchestrators of lower and more ancient regions in the hierarchy of the brain that play a role in survival and are the core components of the Central Autonomic Network (CAN) [11,108,109].

Among this CAN, the most relevant area for descending pain inhibition is the PAG [70,110]. This large and prominent structure of the midbrain (it accounts for approximately 10% of it) is similar across all mammalian brains, including all the animal species widely used as animal models for pain [108,111]. It plays an important role in maintaining the homeostatic balance (allostasis) of the autonomic nervous system, and it also primes basic primitive survival reactions such as fight or flight responses or freezing reactions (i.e., high full-body inhibitory reactions to threats). Based on this, the PAG is positioned as the main source of modulatory inputs for areas such as the LC or the rostroventral medulla (RVM) [25,77].

When a nociceptive signal, collected by the free endings of Aδ, Aβ and the C fibres, ascends through the spine and reaches the thalamus, it has been transduced, modulated, decussated and codified by different relay stations, such as the RVM, PAG, dorsal raphe or the ventral tegmental area [11,63,112]. In theory, each of the relay regions of the ascending nociceptive pathway is subject to modulation by SE or NE pathways, but decades of research on acute and chronic pain animal models has confirmed that most of the descending NE pain inhibition occurs at the posterior horn of the spine, between the first- and the second-order neurons [25,113].

Once the NE is released, it binds to an exclusive NE transporter protein encoded by the gene SLC6A2 and descends through the dorsolateral column of the spine towards the dorsal horn of the spine (mainly cervical and lumbar levels) [18,61,114]. Finally, in conjunction with Dopamine and SE, NE modulates the transmission of pain through three different mechanisms: depolarizing inhibitory GABAergic neurons at the lamina I-III by activation of α1 adrenergic receptors; inhibiting excitatory neurons at the dorsal horn that facilitate nociceptive transmission via α2 adrenergic receptors; or inhibiting the first- and second-order neurons pre- or post-synaptically also via α2 adrenergic receptors [11,17,18]. This descending pathway is frequently used by drugs prescribed for the management of chronic pain, such as gabapentinoids, pregabalin or SE and NE reuptake inhibitors. For an in-depth review of this matter and novel targeted treatments, the reader is referred to the following references [115,116,117,118,119].

Nociceptive signals flag potential internal and external threats that, under non-pathological conditions, could be inhibited in pursuit of survival and appropriate interaction with the environment [120,121]. The system that facilitates this inhibition is a conjunction of complex intricate descending tracts that stem from phylogenetically old autonomic nervous system regions (PAG, LC, RVM, etc.). They modulate ascending signals through LC-NE-mediated circuits, and their correct functioning will guarantee arousal and adequate adaptation to the environment. On the contrary, when the system is overloaded, or its orchestrators and conductors malfunction, the perception of pain is enhanced and, therefore, the individual is exposed to an increased pain perception or chronic pain and the consequent allostatic load. 

## 5. LC and Chronic Pain Comorbidities

**Figure 3:**
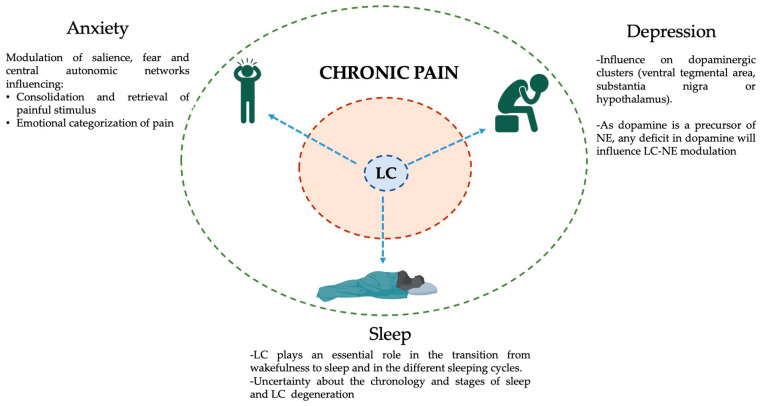
Schematic representation of the influence of the LC in chronic pain and its frequent comorbidities. The inner brown ring represents the intrinsic symptoms of chronic pain: Myofascial pain, aches, soreness, fatigue, allodynia, enhanced somatosensory perception, central sensitization, etc. The outer green ring represents the most frequent chronic pain comorbidities (clockwise → depression, sleep, and anxiety disorders) and the potential effects of the LC on each of them (blue dashed arrows). The existence of a common hub for at least a portion of chronic pain comorbidities will facilitate the development of adapted and individualized clinical responses. This figure was created using PowerPoint (version 16.87) and BioRender 2024.

Given the multifunctional neuro-modulatory role of the LC, it is essential to integrate its many functions within the neurophysiological strata of the frequent mental and physical comorbidities of chronic pain. The following section depicts the potential role of LC in the establishment and perpetuation of three of the most frequent chronic pain comorbidities: anxiety, depression and sleep disorders [4]. Figure 3 depicts the role of LC within a model of chronic pain and its comorbidities.

### 5.1. Anxiety

Anxiety is an emotion of fear or worry about a specific situation that frequently occurs on a continuum with stress [122]. It has a prevalence of 4% across the globe and ranges between 35 and 75% among the chronic pain population [4,123]. Concerning chronic pain, anxiety is a risk factor, or a consequence, and its neural correlates are an excessive activation of the salience, fear and central autonomic and emotion processing networks [109,124].

The LC, through the modulation of these networks, influences memory retrieval and consolidation of the painful stimulus, via its strong projections to the hippocampus; the emotional categorization of the painfulness via modulation of the amygdala; or the modification of pain perception through its connections with the ACC, insula and SSC [124,125,126].

### 5.2. Depression

Even though its prevalence across the globe is very similar to anxiety (around 5%), depression related to chronic pain cannot be considered an emotion, as was the anxiety case, but a mental disorder on its own [127]. It has been shown that 16.4% of chronic pain patients will develop a major depression disorder within 2 years of their diagnosis, and its prevalence increases pain severity [128]. In recent years, it has been pointed out that depression and chronic pain share considerable overlaps in their dopamine-driven neurophysiological changes [129,130]. Moreover, both chronic pain and depression patients suffer from the relocation of attentional resources (lack of concentration, interoceptive focus, etc.) and that has been shown to alter sensory processing across different domains [131,132,133]. This is potentially one of the reasons why the clinical use of antidepressants (especially Duloxetine) has shown great potential for the management of chronic pain in the short-medium term [129,130].

The LC has also been linked to the pathogenesis of depression, and most of its influence across different brain networks relies on dopamine, the precursor of NE [59,129,134]. Dysregulation of the dopamine system is a frequent presentation among multifactorial depressive disorders [135]. Dopaminergic neurons are relatively scarce in the human brain (around 400,000) and they cluster in well-known areas that include the ventral tegmental area, the substantia nigra or the hypothalamus [136,137]. Through the cross-talk between the LC and these three main dopamine hubs, the LC modulates the neurophysiology of chronic pain depressive-related symptoms [68]. Moreover, recent research in mice has identified the mechanisms by which the paraventricular thalamic nucleus generates chronic-pain-associated depressive behaviours [138]. This is particularly interesting because of the already-known strong connections between the LC and this specific thalamic nucleus [139,140]. Further research in humans is needed to comprehend the complex intricate connections that might connect LC to depressive disorders associated with chronic pain.

### 5.3. Sleep Disorders

Sleep deprivation is another frequent comorbidity of chronic pain sufferers and, as it occurs with anxiety and depression, it is difficult to elucidate if it is the cause, consequence or potentially both at the same time [141,142,143]. The prevalence of sleep disorders (i.e., insomnia, difficulty falling asleep or low sleep quality) among the general population is approximately 18.1%, whereas it can reach 65% within the chronic pain cohort [143,144] Interestingly, the more time people suffer from insomnia, the higher the risk of developing chronic pain. However, an improvement in sleeping habits has been associated with a more favourable prognosis [141,144]. 

The recent development of genetically encoded fluorescent sensors for NE has facilitated the tracking of LC-NE release during sleep [145]. The LC plays an essential role both in the transition from wakefulness into sleep and in the transition among the different phases of the sleeping cycles (REM or NREM), with almost null activity during the REM phase [21,146]. Recently, LC-NE neurons have been directly linked with a bidirectional connection between nitroglycerin-induced migraines and acute sleep disturbances [147]. Moreover, it has been proposed that sleep disruption caused by a damaged LC-NE system impairs other functions, like brain–body coordination, neuroplasticity, memory consolidation, neuroinflammation and accelerated levels of neurodegeneration [148,149].

In short, a faulty LC-NE system will facilitate the development of sleep disorders that, if perpetuated in time, impair other pain perceptions. Further research is needed to understand the chronological presentation of sleeping disorders among the chronic pain population and, if possible, how to reverse them.

## 6. LC as a Switch from Acute to Chronic Pain

So far, it has been shown that the descending noradrenergic inhibitory system plays a key role in the automatic control of ascending nociceptive signals. In the absence of pathology, the system is automatic and unspecific and balances nociceptive inputs aiming to maintain homeostasis [25,63]. Therefore, the perpetuation of pain-free states depends on a balance between inputs (medial and lateral pathways) and inhibition (descending pathway). When the nociceptive input is perpetuated for a sufficient amount of time, or there are genetic and/or developmental elements in play, there is a higher risk of developing chronic pain [14,25,150].

Time is an essential element for the neuroplastic changes that need to occur for the central nervous system to facilitate and perpetuate chronic pain. This has been observed in animal models of inflammatory and neuropathic pain, where the analgesic effects provided by the electrical stimulation of the LC reduce by the time the animal is exposed to a nociceptive signal [24,63]. Clinically, in humans, the dysfunction of the inhibitory pain system manifests with a reduction in conditioned pain modulation, and it has been widely observed in primary and secondary chronic pain sufferers [14,113].

This review has shown how the LC plays a role in the modulation and inhibition of ascending nociceptive signals. However, recent research has suggested that the LC might act as a pain inhibitor under healthy conditions and as a pain facilitator if the nociceptive input is sustained in time [24,25]. Based on this research, the LC is influenced both by descending projections from the PFC and insula and/or by excessive ascending inputs from the dorsal horn of the spinal cord. In both cases, the dysfunction is believed to have different mechanisms of action, including glial changes, α2 adrenergic receptors saturation and alterations in firing rates (decreased tonic activity and phasic bursts of the LC), together with other central sensitization and neuroinflammatory changes [24,63].

Another possibility is that the LC facilitates pain and sensory processing through its ascending projections into the ACC and the insula. As has been observed with laminectomies, animal models and neuroimaging, the ACC plays an essential role in aversive responses to painful stimuli [151]. It has also been reported that mice exposed to the perception of pain would show activity in the LC-NE modulatory pathway, reducing the ascending inputs from the spine while enhancing the role of ACC in sensorial processing [23,107,151]. The different distribution of adrenoreceptors might facilitate the saturation of the high-affinity dorsal horn α2 adrenergic receptors but not the low-affinity β-adrenergic receptors present in the cortex and the glial cells (see Table 1) [66]. This will consequently promote both the transmission of ascending nociceptive inputs and cortex sensory processing, explaining common chronic pain clinical findings such as allodynia, general stiffness and soreness.

## 7. Limitations

Narrative reviews provide state-of-the-art updates and evidence-based opinions on specific relevant topics. With this narrative, our goal was to provide insights on advancing the field, current evidence on the role of the LC in chronic pain and lay the groundwork for future research. However, the lack of general guidelines such as Preferred Reporting Items for Systematic Reviews and Meta-Analyses (PRISMA) presents limitations and selection biases that need to be considered when reading this manuscript.

## 8. Conclusion Remarks

The LC is one of the main neuro-modulatory hubs across different brain regions and domains. Its modulatory capacity emerges from its electrical activity (phasic or tonic), neurophysiological properties (NE, dopamine, GABA and serotonin) and macro- and microarchitecture. An impaired LC-NE system reduces the ability of the body to inhibit nociceptive ascending signals while enhancing contextual pain categorisation at higher hierarchical levels such as the ACC and the anterior insula [1,2]. The observed LC functional changes could be explained by factors, such as adrenoreceptor saturation, glial-driven neuroplasticity and descending influences, from areas like the medial PFC, the ACC or other regions involved in emotion, memory or anxiety, such as the hypothalamus, the insular cortex or the amygdala. Future research needs to confirm the fundamental role of LC in chronic pain generation in humans. New animal models, novel fMRI sequences and recent advances in organoids are expected to facilitate that research.

## Figures and Tables

**Table 1:** Represents the current understanding of the adrenoreceptors distribution and their respective NE affinity. (++ represents areas where the concentration is particularly high) (based on [63,64,65].

RECEPTOR	REGIONS FOUND	NE Affinity
α 1 A, B, D	LC, olfactory bulb, cerebral cortex, dentate gyrus, amygdala, thalamus	Intermediate
α 2 A, B, C, D	Pre- and post-synaptically at LC, Amygdala, hypothalamus, spinal cord	High
β 1	Cerebral cortex (astrocytes ++), spinal cord	Low
β 2	Cerebral cortex, cerebellum (astrocytes ++)	Low
β 3	Hippocampus (astrocytes ++)	Low
